# Linking De Novo Assembly Results with Long DNA Reads Using the dnaasm-link Application

**DOI:** 10.1155/2019/7847064

**Published:** 2019-04-11

**Authors:** Wiktor Kuśmirek, Wiktor Franus, Robert Nowak

**Affiliations:** Institute of Computer Science, Warsaw University of Technology, Nowowiejska 15/19, 00-665 Warsaw, Poland

## Abstract

Currently, third-generation sequencing techniques, which make it possible to obtain much longer DNA reads compared to the next-generation sequencing technologies, are becoming more and more popular. There are many possibilities for combining data from next-generation and third-generation sequencing. Herein, we present a new application called dnaasm-link for linking contigs, the result of* de novo* assembly of second-generation sequencing data, with long DNA reads. Our tool includes an integrated module to fill gaps with a suitable fragment of an appropriate long DNA read, which improves the consistency of the resulting DNA sequences. This feature is very important, in particular for complex DNA regions. Our implementation is found to outperform other state-of-the-art tools in terms of speed and memory requirements, which may enable its usage for organisms with a large genome, something which is not possible in existing applications. The presented application has many advantages: (i) it significantly optimizes memory and reduces computation time; (ii) it fills gaps with an appropriate fragment of a specified long DNA read; (iii) it reduces the number of spanned and unspanned gaps in existing genome drafts. The application is freely available to all users under GNU Library or Lesser General Public License version 3.0 (LGPLv3). The demo application, Docker image, and source code can be downloaded from project homepage.

## 1. Introduction

High-throughput sequencing devices, called next-generation sequencers, have provided lots of DNA sequences for various organisms. However, a very large number of draft genome sequences are still incomplete. For example, in GenBank, 90% of bacterial genomes are incomplete [1]. In order to improve the consistency and completeness of the draft of reference genomes, which are produced based on short reads obtained from second-generation sequencers, third-generation long read sequencing can be used. Due to this, third-generation sequencing technologies are becoming more popular; for example, in 2018 the* de novo* human genome assembled from only long DNA reads was published [2].

Third-generation sequencing makes it possible to obtain much longer DNA reads compared to second-generation sequencing technologies. However, the error rate in long reads from third-generation devices compared to short DNA reads from second-generation sequencers is significantly higher [3, 4]. Moreover, the cost per sample of third-generation sequencing is higher than second-generation sequencing [5].

An obvious concept of using both types of reads in* de novo* assembly, hybrid assembly is currently being explored [6, 7]. There are many possibilities for combining data from second-generation sequencing and third-generation sequencing. The four most popular are listed below.

1. Long DNA reads could be mapped directly onto the de Bruijn graph, which is built from short DNA reads. Then, dedicated algorithms allow us to resolve some ambiguity in the de Bruijn graph, which can improve the consistency of the resulting DNA sequences. Such an approach is implemented in some* de novo* DNA assemblers for second-generation reads, e.g., Velvet [8], ABySS [9], and SPAdes [10].
2. Long DNA reads could be* de novo* assembled with dedicated assemblers, e.g., Canu [11], Falcon [12], and miniasm [13]. The created DNA sequences can be improved in terms of quality by mapping short DNA reads and correcting assembly errors using Pilon [14] or quiver [15] applications.
3. Short DNA reads could be used to correct long DNA reads, for example, with CoLoRMap [16] or Nanocorr [17] tools. Then, long and corrected DNA reads could be assembled with assemblers for third-generation sequencing data (as depicted in the previous point).
4. Short DNA reads could be* de novo* assembled using assemblers dedicated to second-generation sequencing data (as depicted in point 1). Then, long DNA reads could be used to link the resulting DNA sequences (contigs), for example, with LINKS [18] or SSPACE-LongRead [19] applications.

In this paper, we present a new application called* dnaasm-link* for combining the output of a* de novo* assembler with long DNA reads (point 4 of the above list). Our software contains a module for filling the gaps between contigs with a specified sequence from an appropriate long DNA read. This feature is very important, in particular for complex DNA regions. What is more, our method has a much shorter calculation time as well as much lower memory requirements in comparison to other tools. Significant memory optimization and reduction of computation time may enable the usage of the application for organisms with a large genome, which may be cumbersome or even impossible for existing applications (estimated resources required for scaffolding of a human genome (~3 Gbp): dnaasm-link, 8 h/600GB; LINKS, 2 days/6TB; SSPACE-LongReads, 5 days/130GB).

The presented algorithm was implemented as a new extension of the dnaasm assembler [20]. The demo application, Docker image, and source code are available at the project homepage: http://dnaasm.sourceforge.net.

## 2. Materials and Methods

The presented algorithm efficiently finds and joins adjacent contigs using long reads. The contigs are produced by a* de novo* DNA assembler from short and high quality reads from second-generation sequencers. In our approach, the contigs are created using the de Bruijn graph algorithm implemented in a dnaasm assembler [20]. The new algorithm, called dnaasm-link, checks which contigs have a subsequence similar to a subsequence in a long read, then finds adjacent contigs, calculates the distance between contigs, and fills the gap with a sequence from the appropriate long DNA read. The presented approach and details of implementation are described below.

### 2.1. Finding Adjacent Contigs

The algorithm uses k-mer similarity to find adjacent contigs. This algorithm consists of several stages.

**Figure 1:**
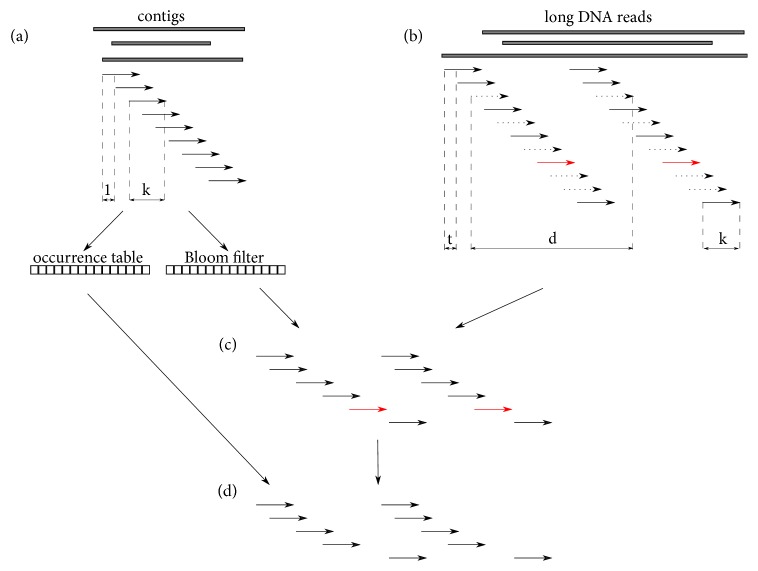
*The process of generating and filtering k-mer pairs from long DNA reads*. (a) Firstly, a Bloom filter and an array containing the number of occurrences of each k-mer are built based on the k-spectrum generated from the input set of contigs. (b) From each long DNA read, a set of k-mer pairs (k-mer length equal to *k*) is generated, with a distance between the beginning of the first k-mer and the end of the second equal to *d* and a sliding step equal to *t*. (c) The input set of k-mer pairs is filtered with the Bloom filter; some pairs are discarded (dotted arrows). (d) The resulting set of k-mer pairs after the second filtering process (red arrows - nonunique k-mers - are discarded). It is worth noting that the resulting set of k-mers pairs (d) is very limited in relation to the generated set of k-mers pairs (b) due to errors in long DNA reads and repetitive regions of the investigated genome.

Firstly, a set of k-mers is generated from the input set of contigs, each of them being inserted into a Bloom filter [21]. A Bloom filter is a probabilistic data structure that efficiently tests whether a k-mer is present in a set. The length of analysed k-mers (the value of parameter *k*) can be set by the user based on the error rate of long DNA reads: the higher the error rate, the lower the *k* value. The default value is 15. This step is depicted in Figure 1(a).

Secondly, a set of long DNA reads begins; a set of k-mer pairs with the distance *d* is generated (paired k-mers that map onto two different contigs are used to link these contigs in the next step of the algorithm). The default value *d* is 4000. It should be mentioned that we do not generate a full k-spectrum here; we rather use the step value *t*, set by default to 2. This step is depicted in Figure 1(b). The pairs in which both k-mers are in the previously generated Bloom filter are processed further, as depicted in Figure 1(c).

Thirdly, a set of unique k-mers is determined. This process consists of counting the number of instances of a given k-mer in the input set of contigs. K-mers which occur more than once are treated as nonunique. All pairs of k-mers containing at least one nonunique k-mer are removed from further considerations, as depicted in Figure 1(d).

Next, a connection graph is built. This graph is composed of vertices that represent contigs and edges that represent connections between contigs derived from pairs of k-mers from long DNA reads. Each edge contains three parameters that define the strength of the specified connection. These parameters are

- the number of connections between a given pair of contigs defined as the number of k-mers pairs;
- the number of connections between a given pair of contigs defined as the number of DNA reads;
- the number of connections between a given pair of contigs defined as the number of DNA reads, where a specified DNA read is taken into consideration if the number of k-mer pairs in this read is greater than the threshold value specified by the user.

After building the connection graph a set of filters is applied to remove some edges representing connections. Filters remove the edges where at least one of the three parameters mentioned above is lower than the corresponding thresholds set by the user.

**Figure 2:**
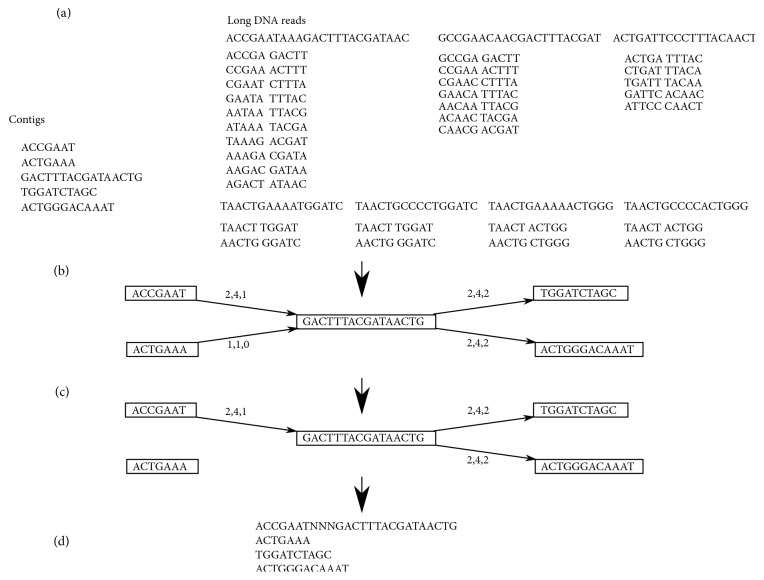
*The process of generating scaffolds from contigs and long DNA reads*. (a) In the presented example there is an input set of contigs composed of five sequences: ACCGAAT, ACTGAAA, GACTTTACGATAACTG, TGGATCTAGC and ACTGGGACAAAT. The set of long reads contains seven sequences: ACCGAATAAAGACTTTACGATAACT, GCCGAACAACGACTTTACGAT, ACTGATTCCCTTTACAACT, TAACTGAAAATGGATC, TAACTGCCCCTGGATC, TAACTGAAAAACTGGG, and TAACTGCCCCACTGGG. Firstly, from each long DNA read a set of k-mer pairs is generated. The values of* k* (k-mer size),* d* (distance between the beginnings of k-mers in a pair), and* t* (sliding step) parameters are equal to 5, 10, and 1, respectively. For example, from the TAACTGAAAATGGATC read, two pairs of k-mers are generated (TAACT,TGGAT) and (AACTG,GGATC). The result of this step is a set of k-mer pairs containing 30 elements. (b) The connection graph built from 30 pairs of k-mers from the previous step and five previously mentioned contigs. Each of the contigs creates a separate vertex. Pairs of k-mers, depending on the contig on which they are located, form the edge of the connection graph. The numbers above the edges represent the number of elements supporting the specified edge, in turn: (i) number of DNA reads, (ii) number of k-mer pairs, and (iii) number of DNA reads where the specified DNA read is taken into account if the number of k-mer pairs in this read is greater than the threshold value (in the presented example the value of this threshold is equal to 1). (c) The filtered connection graph. The applied filter assumes rejection of edges for which there is no DNA read with the number of k-mers above 1 (the third number above the edge should be greater than 0 in proper edges). The values of all three parameters in the filtering step can be set by the user. (d) The result of the algorithm. The set of scaffolds is built from four sequences; the only connection in the example is the combination of ACCGAAT and GACTTTACGATAACTG contigs into the ACCGAATNNNGACTTTACGATAACTG scaffold. This scaffold has not been extended to the right because there is ambiguity of connections. The ratio of the number of k-mer pairs related to the source contig (GACTTTACGATAACTG) is smaller than the threshold value (in the example, the threshold value is equal to 0.3). The ratio, in both cases (GACTTTACGATAACTG with TGGATCTAGC and GACTTTACGATAACTG with ACTGGGACAAAT), is equal to 0.5. It is worth noting that the length of sequence of “N” signs in ACCGAATNNNGACTTTACGATAACTG is equal to 3, which results from the mapping places of the related k-mer pairs, (ACCGA,GACTT), (CCGAA,ACTTT), (CGAAT,CTTTA), and (CCGAA,ACTTT), to the contigs. It is also worth emphasizing that, in gap-filling mode, the “NNN” sequence would be changed to “AAA” from the ACCGAATAAAGACTTTACGATAACT read.

Finally, the process of generating the resulting set of scaffolds from the connection graph is performed. At first, a list of all vertices from the connection graph is prepared. The list should represent contigs sorted by their lengths in descending order. Next, all vertices on the list are marked as unseen. In each iteration an unseen vertex pointing to the longest contig becomes a seed for a new scaffold. The seed is expanded to the right and to the left by attaching consecutive contigs to both ends, based on the connection graph. During the expansion, two situations can occur: (i) the specified contig is connected only with a single vertex in a contig graph, then, considered contigs are joined; (ii) the specified contig is connected with more than a single vertex. In this situation, the vertex with the largest number of connecting pairs of k-mers is preferred. At this stage, two adjacent contigs are not joined into a single sequence, but rather are separated by a gap/overlap placeholder that will be replaced with a proper sequence by the gap-filling algorithm described in the next section. All vertices used to construct a single scaffold are marked as seen and are not taken into consideration in the next iterations of the algorithm. The process is repeated until there are no unseen vertices. The main steps of the described above algorithm are presented in Figure 2.

### 2.2. Gap-Filling Algorithm

When generating scaffolds, two contigs may overlap. In this case, a single “N” sign is inserted between them. However, the contigs may be separated by a gap. The final step of the presented algorithm aims to estimate the gap size and to fill it with a fragment of a long DNA read. For each linking k-mer pair, the gap size is calculated based on (1) the fixed distance between k-mers in the long read, (2) lengths of contigs, and (3) k-mers' offsets on contigs. Sequencing errors in long reads may cause distances computed for each k-mer pair to be different. An average value is taken as an estimate. In the same manner, if the offset of each k-mer pair extracted from the long read is known, it is possible to determine the offset of a subsequence of a read corresponding to each of the gaps in scaffolds. Contigs are covered by reads containing multiple errors, and consequently, multiple different gap sequences may be generated. In the presented application, a gap sequence is taken directly from the read which covers the considered contigs with the greatest number of k-mer pairs.

### 2.3. Implementation

**Figure 3:**
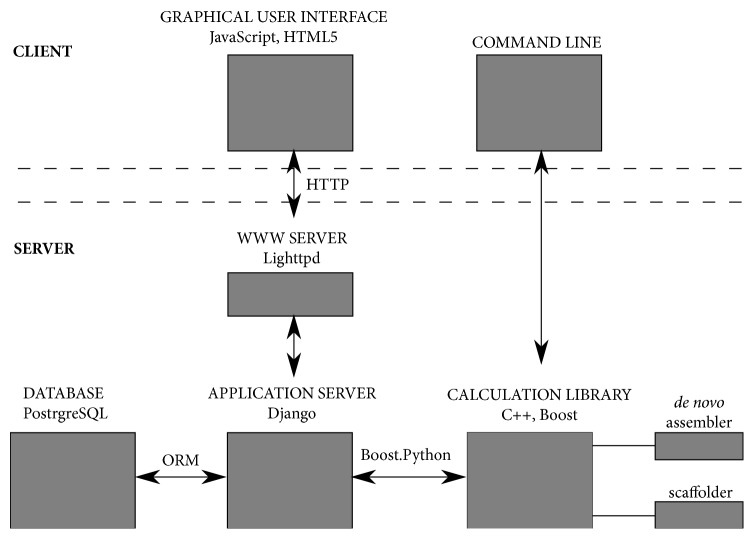
*Architecture of the dnaasm application*. The user can use the application in two ways: through the graphical user interface or a command line. Both ways lead to launching the calculation module in which the presented algorithm is implemented as the shared library. What is more, the calculation module contains an additional shared library in which the* de novo* assembler has been implemented in advance. Both the mentioned assembler and the presented dnaasm-link scaffolder can be launched in a very similar and convenient way.

The dnaasm application was implemented in client-server architecture, based on the bioweb framework [22]. The dnaasm-link is a new module, deployed as a shared library. In our implementation, we used three programming languages: JavaScript, Python, and C++. Firstly, JavaScript along with HTML5 and the AngularJS framework were used to implement the graphical user interface (GUI). Then, the Python and Django library were used to implement the server side. The server side stores parameters of numeric tasks in the PostgreSQL relational database. We decided to use object-relational mapping (ORM) to communicate with the database as this was a flexible and simple approach, and performance issues were not critical. Finally, C++ was used to implement the most complex data processing step, the algorithm presented in the work. Moreover, we used several libraries: Boost, MurmurHash3, and Google Sparse Hash, to make the implementation of our algorithm fast and memory scalable. The main modules of our software are presented in Figure 3.

## 3. Results

Numerical experiments were performed to compare the presented application with other available tools and to indicate the advantages of filling gaps in scaffolds using long DNA reads. Briefly, the first experiment compares the quality of results obtained in the presented method with other tools for hybrid assembly. The second experiment was carried out on artificially generated data and it indicates the benefits of using both short and long DNA reads over using only the output from second-generation sequencers. Finally, the calculation time and memory usage of the application compared to other tools were measured.

To evaluate the quality of resulting DNA sequences in experiments we used QUAST [24] ver. 4.1. We compared DNA sequences in terms of

- the number of resulting DNA sequences longer than 1000 bp;
- the number of misassemblies: sum of relocations, translocations, and inversions;
- N50 statistic: the length of the DNA sequence for which the sum of lengths of all sequences of that length or longer is greater than half of an assembly;
- NA50 statistic: the same as N50, but not for all resulting DNA sequences, only for a set of aligned blocks which are the results of breaking input DNA sequences at misassembly events;
- the largest DNA sequence;
- the largest alignment, the length of the largest continuous alignment in the resulting DNA sequences;
- the average number of mismatches per 100 kbp;
- the average number of indels per 100 kbp;
- the average number of uncalled bases (Ns) per 100 kbp.

Moreover, we used the BUSCO [25] ver. 2.0 tool to compare the DNA sequence in terms of the number of reconstructed core genes, genes present as single-copy in at least 90% of the species from the selected group. As part of the evaluation of DNA sequences, we distinguished four groups: (i) complete and single-copy, (ii) complete and duplicated, (iii) fragmented, and (iv) missing core genes. A detailed description of the experiments and the results obtained can be found in the next parts of this section.

### 3.1. Comparison with Other Tools

We compared the results of our application with other tools for hybrid assembly that connect contigs using long reads. The main objective was comparison in terms of linking the contigs with long DNA reads and filling in the resulting gaps. For the above experiment we used publicly available data for* Escherichia coli* (4,641,652 bp) and* Saccharomyces cerevisiae* (12,157,105 bp). Both of the datasets on which we worked came from Nanocorr's [17] research (http://schatzlab.cshl.edu/data/nanocorr); the names of the files are provided in Supplementary Materials (available here). The above files are the result of* de novo* assembly of short DNA reads and the correction of Oxford Nanopore Technologies (ONT) reads by short DNA reads. Basic parameters of the input set of long DNA reads and contigs are presented in Table 1.

We compared our approach with two state-of-the-art tools used to join contigs into scaffolds with long reads: LINKS [18] ver. 1.8.5 and SSPACE-LongRead [19] ver. 1.1.0. For additional comparison we used scaffolders designed to operate on another kind of read: paired-end tags (PETs) and mate-pairs (MPs). These were OPERA-LG [26] ver. 2.0.6, BOSS [27] and ScaffMatch [28] ver. 0.9.0. We prepared input data for these scaffolders using the Fast-SG [29] tool. This application generates paired DNA reads from long reads and maps such paired reads onto preassembled contigs. Parameter values for the applications and the appropriate commands are provided in Supplementary Materials, while the results of the evaluation are presented in Tables 2 and 3.

Our experiment indicates that the dnaasm-link application gives slightly better results than existing tools in terms of the quantity and quality of the resulting DNA sequences. Looking at N50 and the largest DNA sequence, it seems that dnaasm-link largely improves the assembly. In terms of mismatches and core genes, dnaasm-link seems to be in line with the other approaches. What is more,* de novo* assembly by tools that treat short and long reads differently (LINKS, SSPACE-LongRead, dnaasm-link) gives better results than converting long reads into short reads to increase sequencing coverage followed by* de novo* assembly.

### 3.2. Impact of Adding Long DNA Reads to Contigs Generated from Short DNA Reads

We examined how the combination of short and long DNA reads affects the length and quantity of the resulting DNA sequences. In this study we used the* Saccharomyces cerevisiae* (GenBank NC_001133 … NC_001148, NC_001224) reference genome. From this genome, we generated nine sets of short DNA reads using the pIRS [30] ver. 1.1.1 application and five sets of long reads using the NanoSim [31] ver. 1.0.0 tool, where each set had a different depth of coverage. The details of application used and dataset parameters are provided in Supplementary Materials.

**Figure 4:**
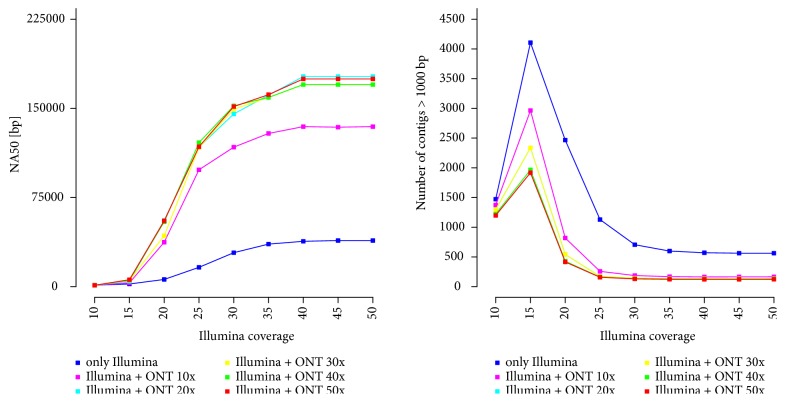
*Impact of adding long DNA reads on the number of resulting scaffolds longer than 1000 bp and the NA50 statistic.* The experiment was conducted on the* Saccharomyces cerevisiae* (GenBank NC_001133 … NC_001148, NC_001224) genome. Firstly, nine sets of short DNA reads and five sets of long DNA reads with different depths of coverage were generated. Then, short reads were* de novo* assembled, and finally, the resulting unitigs were linked by long DNA reads. The peak in number of contigs for Illumina coverage equal to 15x is due to the fact that 10x is too small to cover the whole genome. After increasing the coverage, the number of contigs increases at the beginning, because the whole genome is covered, but with small gaps. It is worth mentioning that a greater depth of coverage does not increase the number of covered gaps in the results, as all the gaps are caused by the complex DNA region and not the lack of coverage. Moreover, the number of contigs obtained only from short reads is greater than the number of sequences after adding long reads. This is because some of the long reads are spread over complex DNA regions; the number of such regions determines the number of contigs [23]. If such a complex region is shorter than a long DNA read, then contigs surrounding it could be joined (with an estimated gap).

The generated short reads were* de novo* assembled by ABySS ver. 2.0.1, then contigs were linked using long reads. The results, presented in Figure 4, prove that combining long DNA reads with short ones can significantly increase the consistency of the resulting assemblies by reducing the final number of scaffolds. Moreover, increasing the coverage of any sequencing technology above a certain level does not improve the results further.

Next, we investigated how the use of long DNA reads affects the reconstruction of complex DNA structures such as long tandem repeats. We compared our method to a technique where gaps are filled with short DNA reads. In this experiment we generated an input set of reads for two organisms:* Escherichia coli* (GenBank NC_000913) and* Saccharomyces cerevisiae* (GenBank NC_001133 … NC_001148, NC_001224). We used the same applications, pIRS and NanoSim as before. Their parameters are provided in Supplementary Materials. The short reads were* de novo* assembled by ABySS [9]. Next, we linked contigs with long DNA reads using the dnaasm-link tool in two modes: with and without gap filling. Then, the scaffolds produced by dnaasm-link without gap filling were treated by three tools for filling gaps with short DNA reads: GapFiller [32] ver. 1.10.0, Sealer [33] ver. 1.9.0, and SOAPdenovo2 GapCloser [34] ver. 1.12.0. Finally, we compared a number of detected tandem repeats using the Tandem Repeats Finder application [35]. This application was also launched on the reference genomes, to determine ground truth data for this study. The results presented in Table 4 depict the advantage of filling gaps using dnaasm-link over other existing methods.

### 3.3. Time and Memory Usage

**Figure 5:**
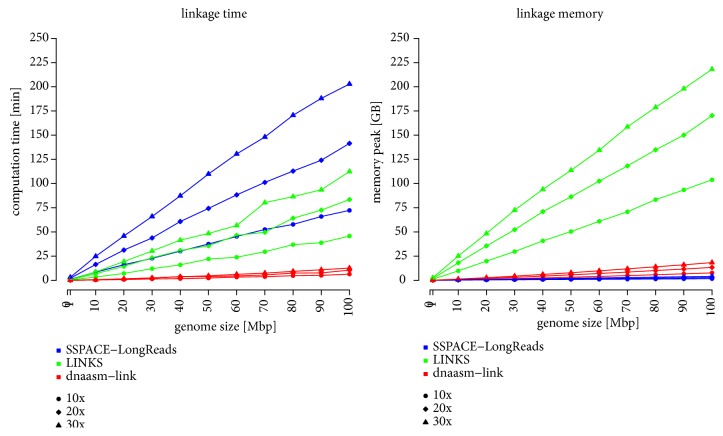
*Comparison of calculation time and peak of RAM memory usage of the SSPACE-LongReads, LINKS and dnaasm-link applications.* The experiment was conducted out on the* Caenorhabditis elegans* genome (GenBank NC_003279 … NC_003284, NC_001328). Firstly, a set of eleven subgenomes of sizes 1Mbp, 10Mbp, 20Mbp … 100Mbp was generated from genome. Then, for each sequence a set of long and short DNA reads was generated. Short DNA reads were* de novo* assembled by ABySS. Finally, the set of resulting contigs and long DNA reads were used as input data sets in the presented experiment.

We examined the dnaasm-link application in terms of performance, as this can be crucial in the analysis of large volume sequencing data. Our application was compared with LINKS [18] and SSPACE-LongRead [19] in terms of time and memory usage. The results are presented in Figure 5.

As expected, combining contigs in applications with accurate mapping takes much more time than in k-mer based tools, in particular, because of the time required to map long DNA reads to preassembled contigs. For example, the calculation time of the SSPACE-LongRead application, for which BLASR [36] software is used in the mapping process, is over 15 times longer than for tools using a k-mer approach, like the dnaasm-link tool. Our tool is significantly faster than the LINKS application, because LINKS, which uses a similar algorithm, is implemented in Perl. In addition, the LINKS application requires much more RAM memory; for example, for a genome of size 100 Mbp and coverage of long reads equal to 30x, the LINKS application uses over 200 GB of RAM memory, and our application only 18.3 GB.

## 4. Discussion

The dnaasm-link application is a new tool for both connecting contigs and filling the gaps between them with long DNA reads. The presented results indicate that the application works similarly to existing tools in terms of the quality of the resulting DNA sequences. However, it works significantly faster with much less RAM memory usage, which can be crucial for large volume sequencing data. Moreover, the presented software contains a module for filling the gaps between contigs with a specified sequence from an appropriate long DNA read, which is not implemented in similar tools.

The procedure of filling the gaps with an appropriate fragment of a specified long DNA read can significantly increase the parameters of the resulting DNA sequences (in the resulting DNA sequences there will be fewer gaps, which may lead to more detailed analyses, e.g., genome annotation). In the presented study we indicated that a very large number of complex DNA structures, especially tandem repeats, could not be properly reproduced without using long DNA reads. Moreover, the addition of long DNA reads, even with very low coverage, can significantly reduce the number of resulting DNA sequences and improve their consistency in relation to the results obtained only from short DNA reads.

In the presented application, a gap within scaffolds could be optionally filled with a fragment of a single long DNA read. However, this solution is not ideal, because such a read may contain many errors, especially if the long reads are raw, i.e., if errors have not been corrected before. In order to control this issue, in the future we plan to add a module to create consensus from several DNA reads. The result of the consensus of several long reads would be inserted into the gap instead of the raw fragment of a single long read, which would significantly reduce the number of errors in the considered DNA fragments. However, the preliminary study shows a big increase in time complexity when consensus is calculated with the use of a multialignment dynamic programming algorithm.

In the future, we also plan to add a module for analysing the similarity of k-mers, which would take into account the fact that the k-mers may contain errors. The presented tool is based on k-mers, which should contain as few errors as possible, because each single error in the specified DNA sequence causes the creation of *k* erroneous k-mers in the k-spectrum. To deal with this problem, in the next version of the software, we will add a module which will investigate the profile of a specified k-mer and compare it to the profiles of other k-mers. The profile will contain several pieces of information, e.g., number of specified 2-mers and their location in the investigated k-mer.

The presented application is available under GNU Library or Lesser General Public License version 3.0 (LGPLv3). In order to easily use the software, the demo application with web interface as well as the Docker [37] container with the dnaasm-link tool is available. What is more, the user can download binary files as well as source code and compile the application with any changes in the algorithm.

## 5. Conclusion

As more and more genomes are sequenced, it becomes desirable to correctly reproduce their DNA sequences, especially, from short and long DNA reads. Here we have presented dnaasm-link, a tool for linking contigs, the result of* de novo* assembly of second-generation sequencing data, with long DNA reads.

## Figures and Tables

**Table 1:** The input set of long DNA reads and contigs characteristic for *E. coli* and *S. cerevisiae* organisms from Nanocorr's research.

		No. of sequences	Sum [Mbp]	N50 [bp]	Max [bp]	Avg. mis.	Avg. indels	Avg. N's
contigs	*E. coli*	65	4.681	176396	398301	2.32	0.17	0.00
	*S. cerevisiae*	430	14.911	53444	257346	85.77	8.80	0.00

long reads	*E. coli*	59009	240.098	7471	43798	180.75	181.20	0.00
	*S. cerevisiae*	88218	526.589	9189	72879	360.98	171.80	5.06

**Table 2:** *Evaluation of dnaasm-link application in comparison to other tools for datasets depicted in Table 1*. The first row in table, where algorithm name is “no scaffolding”, provides the input set statistics (no scaffolding algorithm is used) taken from Table 1. The parameters (No. of contigs, etc.) are depicted in first paragraph of “Results” section.

		No. of contigs	No. of mis.	N50 [bp]	NA50 [bp]	Max [bp]	Largest algn. [bp]	Avg. mis.	Avg. indels	Avg. N's
*E. coli*	no scaffolding	65	9	176396	164044	398301	360084	2.32	0.17	0.00
	SSPACE-LongRead	32	29	398301	211043	1274776	564486	2.47	0.37	570.90
	LINKS	23	19	637611	235726	1146701	636452	2.36	0.39	233.43
	*dnaasm-link *	* 22 *	* 20 *	* 746714 *	* 219242 *	* 1128693 *	* 636452 *	* 2.36 *	* 0.37 *	* 212.75 *
	Fast-SG + OPERA-LG	26	16	349966	342146	659623	658295	2.36	0.30	326.53
	Fast-SG + BOSS	60	14	177523	164044	611106	360084	2.32	0.17	64.79
	Fast-SG + ScaffMatch	55	18	185955	177523	603113	359089	2.41	0.17	139.44

*S. cerevisiae*	no scaffolding	430	53	53444	49075	257346	249232	85.77	8.80	0.00
	SSPACE-LongRead	557	105	167867	126607	736874	452023	95.42	11.27	3690.74
	LINKS	202	89	202618	126598	623140	416048	87.04	10.00	850.77
	* dnaasm-link *	* 190 *	* 92 *	* 224004 *	* 126353 *	* 764024 *	* 431875 *	* 87.28 *	* 10.08 *	* 861.19 *
	Fast-SG + OPERA-LG	202	59	180866	155226	736942	451889	85.51	9.72	462.50
	Fast-SG + BOSS	369	113	57097	47994	257346	249232	85.77	8.80	374.16
	Fast-SG + ScaffMatch	328	144	80833	51157	434320	249232	85.41	8.82	489.70

The following reference sequences were used to evaluate the results: NC_000913 for *E. coli* and NC_001133 … NC_001148, NC_001224 for *S. cerevisiae*.

**Table 3:** Comparison of the number of core genes reproduced from datasets depicted in Table 1.

		Complete and single-copy	Complete and duplicated	Fragmented	Missing
*E. coli*	NGS contigs	780	0	1	0
	SSPACE-LongRead	619	162	0	0
	LINKS	780	0	1	0
	* dnaasm-link *	* 780 *	* 0 *	* 1 *	* 0 *
	Fast-SG + OPERA-LG	780	0	1	0
	Fast-SG + BOSS	780	0	1	0
	Fast-SG + ScaffMatch	780	0	1	0

*S. cerevisiae*	NGS contigs	1657	9	18	27
	SSPACE-LongRead	1647	27	15	22
	LINKS	1661	9	14	27
	* dnaasm-link *	* 1659 *	* 10 *	* 14 *	* 28 *
	Fast-SG + OPERA-LG	1661	9	16	25
	Fast-SG + BOSS	1660	9	18	24
	Fast-SG + ScaffMatch	1658	9	12	32

The sets of reference core genes used for evaluation were enterobacteriales_odb9 (781 core genes) and saccharomycetales_odb9 (1712 core genes) for *E. coli* and *S. cerevisiae*, respectively.

**Table 4:** Tandem repeat reconstruction efficiency.

	Motif len. [bp]	Num of repet.	NGS unitigs	dnaasm-link without gap fill.	dnaasm-link without gap filling			dnaasm-link with gap fill.
					+ GapFiller	+ Sealer	+ GapCloser	
*E. coli*	181	3.0	-	-	-	-	-	-
	181	2.3	-	-	-	-	-	-
	178	1.9	-	-	+	-	-	+
	226	2.0	-	-	-	-	-	+
	113	2.7	-	-	-	-	-	+
	226	1.9	-	-	-	-	-	-
	200	2.0	-	-	-	-	-	+

*S. cerevisiae*	135	1.9	-	-	-	-	-	-
	135	1.9	-	-	-	-	-	-
	135	3.1	-	-	-	-	-	-
	135	3.1	-	-	-	-	-	-
	135	1.9	-	-	-	-	-	-
	192	2.2	-	-	-	-	-	-
	192	2.1	-	-	-	-	-	-
	84	3.0	-	-	-	-	-	-
	1998	2.0	-	-	-	-	-	-
	207	2.1	-	-	-	-	-	+
	81	3.3	-	-	-	-	-	+
	189	1.9	-	-	-	-	-	+
	72	5.3	-	-	-	-	-	+
	189	2.3	-	-	-	-	-	+

The table presents all tandem repeats in the *E. coli* and *S. cerevisiae* reference genomes. In the presented table “+” signs mean the correct reproduction of the specified repetitive fragment and “-” signs mean the lack of correct reconstruction. The presented results indicate that the usage of long DNA reads by dnaasm-link tool allows reconstructing some of tandem repeats.

## Data Availability

dnaasm-link is implemented in C++ and is freely available under GNU Library or Lesser General Public License version 3.0 (LGPLv3). It and related materials can be downloaded from project homepage http://dnaasm.sourceforge.net.

## Supplementary Materials

Supplementary Materials File with commands to reproduce experiments.
