# Supplementary material for: Linking De Novo Assembly Results with Long DNA Reads Using the dnaasm-link Application
Source: Biomed Res Int. 2019 Apr 11;2019:7847064. doi: 10.1155/2019/7847064 (PMC6487145; doi:10.1155/2019/7847064)
Supplement: Supplementary Materials — File with commands to reproduce experiments. [file 7847064.f1.pdf]

Linking *de novo* assembly results with long DNA reads using the dnaasm-link application

Wiktor Kuśmirek, Wiktor Franus, Robert Nowak

Supplementary Materials

1 Comparison with another tools

The used datasets came from Nanocorr's research (http://schatzlab.cshl.edu/data/nanocorr), files:

- ecoli_Miseq_Assembly.fa
- ecoli_ONT_Nanocorr_Corrected_reads.fa
- W303_Miseq_Assembly.fa
- W303_ONT_Nanocorr_Corrected_independent_reads.fa

1.1 Escherichia coli

Firstly, we used SSPACE-LongRead tool:

```
cd SSPACE-LongRead_v1-1
perl SSPACE-LongRead.pl -c schatzlab_coli/input/ecoli_Miseq_Assembly.fa \
  -p schatzlab_coli/input/ecoli_ONT_Nanocorr_Corrected_reads.fa \
  -b results -k 1
mv SSPACE-LongRead_v1-1/results/scaffolds.fasta \
  schatzlab_coli/output/sspace_longreads.fa
cd bbmap
./rename.sh schatzlab_coli/output/sspace_longreads.fa \
  out=out.fa
mv -f out.fa schatzlab_coli/output/sspace_longreads.fa
```

Then, we tested LINKS application:

```
cd links_v1.8.5
echo 'schatzlab_coli/input/ecoli_ONT_Nanocorr_Corrected_reads.fa' \
  > nano_reads
perl LINKS.pl -f schatzlab_coli/input/ecoli_Miseq_Assembly.fa \
  -s nano_reads -z 1
mv schatzlab_coli/input/ecoli_Miseq_Assembly.fa.scaff*.fa \
  schatzlab_coli/output/links.fa
cd bbmap
./rename.sh schatzlab_coli/output/links.fa out=out.fa
mv -f out.fa schatzlab_coli/output/links.fa
```

After that, we generated set of short, paired DNA reads from long reads by Fast-SG:

```
cd fastsg/
echo 'long long_reads \
  schatzlab_coli/input/ecoli_ONT_Nanocorr_Corrected_reads.fa \
  4000 1' > schatzlab_coli/reads.txt
./FAST-SG.pl -k 15 -l schatzlab_coli/reads.txt \
  -r schatzlab_coli/input/ecoli_Miseq_Assembly.fa -p results
cd bbmap/
./splitsam.sh ../fastsg/long_reads.I4000.FastSG_K15.sam \
  ../fastsg/long_reads.I4000.FastSG_K15.FW.sam \
  ../fastsg/long_reads.I4000.FastSG_K15.RV.sam
```

```
cd fastsg/
samtools view -bS long_reads.I4000.FastSG_K15.FW.sam \
  -o long_reads.I4000.FastSG_K15.FW.bam \
  -T schatzlab_coli/input/ecoli_Miseq_Assembly.fa
samtools view -bS long_reads.I4000.FastSG_K15.RV.sam \
  -o long_reads.I4000.FastSG_K15.RV.bam \
  -T schatzlab_coli/input/ecoli_Miseq_Assembly.fa
```

Then, we launched OPERA-LG software:

```
cd OPERA-LG_v2.0.6/bin
printf 'output_folder=results\n \
  contig_file=schatzlab_coli/input/ecoli_Miseq_Assembly.fa\n \
  filter_repeat=yes\nhaploid_coverage=50\n[LIB]\n \
  map_file=fastsg/long_reads.I4000.FastSG_K15.sam' \
  > opera.K15.ont_raw.conf
./OPERA-LG opera.K15.ont_raw.conf
mv results/scaffoldSeq.fasta schatzlab_coli/output/opera-lg.fa
cd bbmap
./rename.sh schatzlab_coli/output/opera-lg.fa out=out.fa
mv -f out.fa schatzlab_coli/output/opera-lg.fa
```

Then, we tested BOSS tool:

```
cd BOSS;
./boss schatzlab_coli/input/ecoli_Miseq_Assembly.fa \
  fastsg/long_reads.I4000.FastSG_K15.FW.bam \
  fastsg/long_reads.I4000.FastSG_K15.RV.bam \
  75 4115 0.1 0.2 5 1 0 results;
mv results_ScaffoldSet.fa schatzlab_coli/output/boss.fa;
```

Then, we used ScaffMatch application:

```
cd ScaffMatch
./scaffmatch -0.9 -m -w results \
  -c schatzlab_coli/input/ecoli_Miseq_Assembly.fa \
  -1 fastsg/long_reads.I4000.FastSG_K15.FW.sam \
  -2 fastsg/long_reads.I4000.FastSG_K15.RV.sam \
  -i 4115 -p fr -s 400 -t 5
mv results/scaffolds.fa schatzlab_coli/output/scaffmatch.fa
```

Lastly, we used dnaasm-link tool:

```
cd dnaasm
./dnaasm -scaffold \
  -contigs_file_path schatzlab_coli/input/ecoli_Miseq_Assembly.fa \
  -long_reads_file_path \
  schatzlab_coli/input/ecoli_ONT_Nanocorr_Corrected_reads.fa \
  -min_contig_length 1
mv dnaasm/out schatzlab_coli/output/dnaasm.fa
cd bbmap
./rename.sh schatzlab_coli/output/dnaasm.fa out=out.fa
mv -f out.fa schatzlab_coli/output/dnaasm.fa
```

Finally, we evaluated resultant DNA sequences:

```
cd quast-4.1
./quast.py schatzlab_coli/input/ecoli_Miseq_Assembly.fa \
  -R schatzlab_coli/ref.fa --scaffolds
./quast.py schatzlab_coli/output/sspace_longreads.fa \
  -R schatzlab_coli/ref.fa --scaffolds
./quast.py schatzlab_coli/output/links.fa \
  -R schatzlab_coli/ref.fa --scaffolds
./quast.py schatzlab_coli/output/opera-lg.fa \
  -R schatzlab_coli/ref.fa --scaffolds
./quast.py schatzlab_coli/output/boss.fa \
  -R schatzlab_coli/ref.fa --scaffolds
./quast.py schatzlab_coli/output/scaffmatch.fa \
  -R schatzlab_coli/ref.fa --scaffolds
./quast.py schatzlab_coli/output/dnaasm.fa \
  -R schatzlab_coli/ref.fa --scaffolds

./busco -f -c 16 -o a -i schatzlab_coli/input/ecoli_Miseq_Assembly.fa \
  -l enterobacteriales_odb9 -m genome
./busco -f -c 16 -o a -i schatzlab_coli/output/sspace_longreads.fa \
  -l enterobacteriales_odb9 -m genome
./busco -f -c 16 -o a -i schatzlab_coli/output/links.fa \
  -l enterobacteriales_odb9 -m genome
./busco -f -c 16 -o a -i schatzlab_coli/output/opera-lg.fa \
  -l enterobacteriales_odb9 -m genome
./busco -f -c 16 -o a -i schatzlab_coli/output/boss.fa \
  -l enterobacteriales_odb9 -m genome
./busco -f -c 16 -o a -i schatzlab_coli/output/scaffmatch.fa \
  -l enterobacteriales_odb9 -m genome
./busco -f -c 16 -o a -i schatzlab_coli/output/dnaasm.fa \
  -l enterobacteriales_odb9 -m genome
```

1.2 Saccharomyces cerevisiae

Firstly, we used SSPACE-LongRead tool:

```
cd SSPACE-LongRead_v1-1
perl SSPACE-LongRead.pl
  -c schatzlab_yeast/input/W303_Miseq_Assembly.fa \
  -p schatzlab_yeast/input/W303_ONT_Nanocorr_Corrected_independent_reads.fa\
  -b results -k 1
mv SSPACE-LongRead_v1-1/results/scaffolds.fasta \
  schatzlab_yeast/output/sspace_longreads.fa
```

Then, we tested LINKS application:

```
cd links_v1.8.5
echo 'schatzlab_yeast/input/W303_Nanocorr_Corrected_independent_reads.fa' \
  > nano_reads
```

```
perl LINKS.pl -f schatzlab_yeast/input/W303_Miseq_Assembly.fa \
  -s nano_reads -z 1
mv schatzlab_yeast/input/W303_Miseq_Assembly.fa.scaff*.fa \
  schatzlab_yeast/output/links.fa
cd bbmap
./rename.sh schatzlab_yeast/output/links.fa out=out.fa
mv -f out.fa schatzlab_yeast/output/links.fa
```

After that, we generated set of short, paired DNA reads from long reads by Fast-SG:

```
cd fastsg/
echo 'long long_reads \
  schatzlab_yeast/input/W303_Nanocorr_Corrected_independent_reads.fa 4000 1'
  > schatzlab_yeast/reads.txt
./FAST-SG.pl -k 15 -l schatzlab_yeast/reads.txt \
  -r schatzlab_yeast/input/W303_Miseq_Assembly.fa \
  -p results
cd bbmap/
./splitsam.sh ../fastsg/long_reads.I4000.FastSG_K15.sam \
  ../fastsg/long_reads.I4000.FastSG_K15.FW.sam \
  ../fastsg/long_reads.I4000.FastSG_K15.RV.sam
cd fastsg/
samtools view -bS long_reads.I4000.FastSG_K15.FW.sam \
  -o long_reads.I4000.FastSG_K15.FW.bam \
  -T schatzlab_yeast/input/W303_Miseq_Assembly.fa
samtools view -bS long_reads.I4000.FastSG_K15.RV.sam \
  -o long_reads.I4000.FastSG_K15.RV.bam \
  -T schatzlab_yeast/input/W303_Miseq_Assembly.fa
```

Then, we launched OPERA-LG software:

```
cd OPERA-LG_v2.0.6/bin
printf 'output_folder=results\n \
  contig_file=schatzlab_yeast/input/W303_Miseq_Assembly.fa\n \
  filter_repeat=yes\nhaploid_coverage=50\n[LIB]\n \
  map_file=fastsg/long_reads.I4000.FastSG_K15.sam' > opera.K15.ont_raw.conf
./OPERA-LG opera.K15.ont_raw.conf
mv results/scaffoldSeq.fasta schatzlab_yeast/output/opera-lg.fa
cd bbmap
./rename.sh schatzlab_yeast/output/opera-lg.fa out=out.fa
mv -f out.fa schatzlab_yeast/output/opera-lg.fa
```

Then, we tested BOSS tool:

```
cd BOSS;
./boss schatzlab_yeast/input/W303_Miseq_Assembly.fa \
  fastsg/long_reads.I4000.FastSG_K15.FW.bam \
  fastsg/long_reads.I4000.FastSG_K15.RV.bam \
  75 4115 0.1 0.2 5 1 0 results;
mv results_ScaffoldSet.fa schatzlab_yeast/output/boss.fa;
```

Then, we used ScaffMatch application:

```
cd ScaffMatch
./scaffmatch -0.9 -m -w results \
  -c schatzlab_yeast/input/W303_Miseq_Assembly.fa \
  -1 fastsg/long_reads.I4000.FastSG_K15.FW.sam \
  -2 fastsg/long_reads.I4000.FastSG_K15.RV.sam \
  -i 4115 -p fr -s 400
mv results/scaffolds.fa schatzlab_yeast/output/scaffmatch.fa
```

Lastly, we used dnaasm-link tool:

```
cd dnaasm
./dnaasm -scaffold -contigs_file_path \
  schatzlab_yeast/input/W303_Miseq_Assembly.fa -long_reads_file_path \
  schatzlab_yeast/input/W303_ONT_Nanocorr_Corrected_independent_reads.fa \
  -min_contig_length 1
mv dnaasm/out schatzlab_yeast/output/dnaasm.fa
cd bbmap
./rename.sh schatzlab_yeast/output/dnaasm.fa out=out.fa
mv -f out.fa schatzlab_yeast/output/dnaasm.fa
```

Finally, we evaluated resultant DNA sequences:

```
cd quast-4.1
./quast.py schatzlab_yeast/input/W303_Miseq_Assembly.fa \
  -R schatzlab_yeast/ref.fa --scaffolds
./quast.py schatzlab_yeast/output/sspace_longreads.fa \
  -R schatzlab_yeast/ref.fa --scaffolds
./quast.py schatzlab_yeast/output/links.fa \
  -R schatzlab_yeast/ref.fa --scaffolds
./quast.py schatzlab_yeast/output/opera-lg.fa \
  -R schatzlab_yeast/ref.fa --scaffolds
./quast.py schatzlab_yeast/output/boss.fa \
  -R schatzlab_yeast/ref.fa --scaffolds
./quast.py schatzlab_yeast/output/scaffmatch.fa \
  -R schatzlab_yeast/ref.fa --scaffolds
./quast.py schatzlab_yeast/output/dnaasm.fa \
  -R schatzlab_yeast/ref.fa --scaffolds

./busco -f -c 16 -o a -i schatzlab_yeast/input/W303_Miseq_Assembly.fa \
  -l saccharomycetales_odb9 -m genome
./busco -f -c 16 -o a -i schatzlab_yeast/output/sspace_longreads.fa \
  -l saccharomycetales_odb9 -m genome
./busco -f -c 16 -o a -i schatzlab_yeast/output/links.fa \
  -l saccharomycetales_odb9 -m genome
./busco -f -c 16 -o a -i schatzlab_yeast/output/opera-lg.fa \
  -l saccharomycetales_odb9 -m genome
./busco -f -c 16 -o a -i schatzlab_yeast/output/boss.fa \
  -l saccharomycetales_odb9 -m genome
./busco -f -c 16 -o a -i schatzlab_yeast/output/scaffmatch.fa \
  -l saccharomycetales_odb9 -m genome
./busco -f -c 16 -o a -i schatzlab_yeast/output/dnaasm.fa \
```

```
  -l saccharomycetales_odb9 -m genome
```

2 The impact of adding long DNA reads

2.1 Mixing short and long reads - NA50 and number of contigs longer than 1 kbp

Firstly, we generated set of short reads by pIRS application:

```
cd pIRS_111
./pirs simulate -i ref.fa -e 0.01 -a 1 -o test -x 10 -m 400 -v 40
mv test_100_400_* 10_0/input/illumina/
```

Then, we generated set of long reads by NanoSim application:

```
cd NanoSim/src
./simulator.py linear -r ref.fa -c ecoli -o simulated -n 24250 \
  --min_len 1000 --max_len 10000
mv simulated_reads.fasta 10_10/input/nanopore
```

After that, we assembled set of short reads by ABySS tool and removed unitigs shorter than 1 kbp:

```
cd abyss
abyss-pe name=org k=55 in='10_0/input/illumina/test_100_400_1.fq.gz \
  10_0/input/illumina/test_100_400_2.fq.gz'
mv org-3.fa 10_0/output/abyssUnitigsFromPET.fa
cd 10_0/output/
python fastaLengthFilter.py abyssUnitigsFromPET.fa 1000 100000000 \
  abyssUnitigsFromPET_1000.fa
```

Lastly, we linked contigs by LINKS tool:

```
cd links_v1.8.5
echo '10_10/input/nanopore/simulated_reads.fasta' > nano_reads
perl LINKS.pl -f 10_0/output/abyssUnitigsFromPET_1000.fa \
  -s nano_reads -z 1 -l 0 -a 0.01
mv 10_0/output/abyssUnitigsFromPET_1000.fa.scaff_s-*.scaffolds.fa \
  10_10/output/links_from_unitigs_perfect_ont.fa
```

Finally, we evaluated resultant DNA sequences:

```
cd quast-4.1
./quast.py 10_10/output/links_from_unitigs_perfect_ont.fa \
  -R ref.fa --scaffolds
less quast_results/latest/report.txt
```

2.2 Mixing short and long reads - tandem repeats

2.2.1 *Escherichia coli*

Firstly, we generated set of short reads by pIRS application:

```
cd pIRS_111
./pirs simulate -i Esc_col/ref.fa -e 0.01 -a 1 -o test -x 50 -m 400 -v 40
mv test_100_400_* Esc_col/input/illumina/
```

Then, we generated set of long reads by NanoSim application:

```
cd NanoSim/src
./simulator.py linear -r Esc_col/ref.fa -c ecoli -o simulated -n 18400 \
  --min_len 1000 --max_len 10000 --perfect
mv simulated_reads.fasta Esc_col/input/nanopore
```

After that, we assembled set of short reads by ABySS tool and removed unitigs shorter than 1 kbp:

```
cd abyss
abyss-pe name=org k=55 in='Esc_col/input/illumina/test_100_400_1.fq.gz \
  Esc_col/input/illumina/test_100_400_2.fq.gz'
mv org-3.fa Esc_col/output/abyssUnitigsFromPET.fa
cd Esc_col/output/
python fastaLengthFilter.py abyssUnitigsFromPET.fa 1000 100000000 \
  abyssUnitigsFromPET_1000.fa
```

Then, we linked contigs by dnaasm-link tool without gap filling:

```
./dnaasm -scaffold \
  -contigs_file_path Esc_col/output/abyssUnitigsFromPET_1000.fa \
  -long_reads_file_path Esc_col/input/nanopore/simulated_reads.fasta \
  -min_contig_length 0 -gapfilling 0 -min_reads 5 -min_lpr 5 -max_ratio 0.3\
  -kmer_size 15 -min_links 5 -distance 1000 -step 1
mv out out_1000
./dnaasm -scaffold -contigs_file_path out_1000 \
  -long_reads_file_path Esc_col/input/nanopore/simulated_reads.fasta \
  -min_contig_length 0 -gapfilling 0 -min_reads 5 -min_lpr 5 -max_ratio 0.3\
  -kmer_size 15 -min_links 5 -distance 2000 -step 1
mv out out_2000
./dnaasm -scaffold -contigs_file_path out_2000 \
  -long_reads_file_path Esc_col/input/nanopore/simulated_reads.fasta \
  -min_contig_length 0 -gapfilling 0 -min_reads 5 -min_lpr 5 -max_ratio 0.3\
  -kmer_size 15 -min_links 5 -distance 3000 -step 1
mv out out_3000
./dnaasm -scaffold -contigs_file_path out_3000 \
  -long_reads_file_path Esc_col/input/nanopore/simulated_reads.fasta \
  -min_contig_length 0 -gapfilling 0 -min_reads 5 -min_lpr 5 -max_ratio 0.3\
  -kmer_size 15 -min_links 5 -distance 4000 -step 1
mv out out_4000
./dnaasm -scaffold -contigs_file_path out_4000 \
  -long_reads_file_path Esc_col/input/nanopore/simulated_reads.fasta \
  -min_contig_length 0 -gapfilling 0 -min_reads 5 -min_lpr 5 -max_ratio 0.3\
  -kmer_size 15 -min_links 5 -distance 5000 -step 1
mv out out_5000
./dnaasm -scaffold -contigs_file_path out_5000 \
  -long_reads_file_path Esc_col/input/nanopore/simulated_reads.fasta \
  -min_contig_length 0 -gapfilling 0 -min_reads 5 -min_lpr 5 -max_ratio 0.3\
  -kmer_size 15 -min_links 5 -distance 6000 -step 1
mv out out_6000
```

```
./dnaasm -scaffold -contigs_file_path out_6000 \
  -long_reads_file_path Esc_col/input/nanopore/simulated_reads.fasta \
  -min_contig_length 0 -gapfilling 0 -min_reads 5 -min_lpr 5 -max_ratio 0.3\
  -kmer_size 15 -min_links 5 -distance 7000 -step 1
mv out out_7000
./dnaasm -scaffold -contigs_file_path out_7000 \
  -long_reads_file_path Esc_col/input/nanopore/simulated_reads.fasta \
  -min_contig_length 0 -gapfilling 0 -min_reads 5 -min_lpr 5 -max_ratio 0.3\
  -kmer_size 15 -min_links 5 -distance 8000 -step 1
mv out out_8000
./dnaasm -scaffold -contigs_file_path out_8000 \
  -long_reads_file_path Esc_col/input/nanopore/simulated_reads.fasta \
  -min_contig_length 0 -gapfilling 0 -min_reads 5 -min_lpr 5 -max_ratio 0.3\
  -kmer_size 15 -min_links 5 -distance 9000 -step 1
mv out out_9000
./dnaasm -scaffold -contigs_file_path out_9000 \
  -long_reads_file_path Esc_col/input/nanopore/simulated_reads.fasta \
  -min_contig_length 0 -gapfilling 0 -min_reads 5 -min_lpr 5 -max_ratio 0.3\
  -kmer_size 15 -min_links 5 -distance 10000 -step 1
bbmap/rename.sh in=out out=out.fa
mv out.fa Esc_col/output/dnaasm_without_gapfilling_from_unitigs.fa
```

Then, we linked contigs by dnaasm-link tool with gap filling:

```
./dnaasm -scaffold \
  -contigs_file_path Esc_col/output/abyssUnitigsFromPET_1000.fa \
  -long_reads_file_path Esc_col/input/nanopore/simulated_reads.fasta \
  -min_contig_length 0 -gapfilling 1 -min_reads 5 -min_lpr 5 -max_ratio 0.3\
  -kmer_size 15 -min_links 5 -distance 1000 -step 1
mv out out_1000
./dnaasm -scaffold -contigs_file_path out_1000 \
  -long_reads_file_path Esc_col/input/nanopore/simulated_reads.fasta \
  -min_contig_length 0 -gapfilling 1 -min_reads 5 -min_lpr 5 -max_ratio 0.3\
  -kmer_size 15 -min_links 5 -distance 2000 -step 1
mv out out_2000
./dnaasm -scaffold -contigs_file_path out_2000 \
  -long_reads_file_path Esc_col/input/nanopore/simulated_reads.fasta \
  -min_contig_length 0 -gapfilling 1 -min_reads 5 -min_lpr 5 -max_ratio 0.3\
  -kmer_size 15 -min_links 5 -distance 3000 -step 1
mv out out_3000
./dnaasm -scaffold -contigs_file_path out_3000 \
  -long_reads_file_path Esc_col/input/nanopore/simulated_reads.fasta \
  -min_contig_length 0 -gapfilling 1 -min_reads 5 -min_lpr 5 -max_ratio 0.3\
  -kmer_size 15 -min_links 5 -distance 4000 -step 1
mv out out_4000
./dnaasm -scaffold -contigs_file_path out_4000 \
  -long_reads_file_path Esc_col/input/nanopore/simulated_reads.fasta \
  -min_contig_length 0 -gapfilling 1 -min_reads 5 -min_lpr 5 -max_ratio 0.3\
  -kmer_size 15 -min_links 5 -distance 5000 -step 1
mv out out_5000
```

```
./dnaasm -scaffold -contigs_file_path out_5000 \
  -long_reads_file_path Esc_col/input/nanopore/simulated_reads.fasta \
  -min_contig_length 0 -gapfilling 1 -min_reads 5 -min_lpr 5 -max_ratio 0.3\
  -kmer_size 15 -min_links 5 -distance 6000 -step 1
mv out out_6000
./dnaasm -scaffold -contigs_file_path out_6000 \
  -long_reads_file_path Esc_col/input/nanopore/simulated_reads.fasta \
  -min_contig_length 0 -gapfilling 1 -min_reads 5 -min_lpr 5 -max_ratio 0.3\
  -kmer_size 15 -min_links 5 -distance 7000 -step 1
mv out out_7000
./dnaasm -scaffold -contigs_file_path out_7000 \
  -long_reads_file_path Esc_col/input/nanopore/simulated_reads.fasta \
  -min_contig_length 0 -gapfilling 1 -min_reads 5 -min_lpr 5 -max_ratio 0.3\
  -kmer_size 15 -min_links 5 -distance 8000 -step 1
mv out out_8000
./dnaasm -scaffold -contigs_file_path out_8000 \
  -long_reads_file_path Esc_col/input/nanopore/simulated_reads.fasta \
  -min_contig_length 0 -gapfilling 1 -min_reads 5 -min_lpr 5 -max_ratio 0.3\
  -kmer_size 15 -min_links 5 -distance 9000 -step 1
mv out out_9000
./dnaasm -scaffold -contigs_file_path out_9000 \
  -long_reads_file_path Esc_col/input/nanopore/simulated_reads.fasta \
  -min_contig_length 0 -gapfilling 1 -min_reads 5 -min_lpr 5 -max_ratio 0.3\
  -kmer_size 15 -min_links 5 -distance 10000 -step 1
bbmap/rename.sh in=out out=out.fa
mv out.fa Esc_col/output/dnaasm_with_gapfilling_from_unitigs.fa
```

Then, we filled gaps by GapFiller, Sealer and SOAPdenovo2 GapCloser tools:

```
cd GapFiller_v1-10_linux-x86_64
printf "lib_1␣bwa␣Esc_col/input/illumina/test_100_400_1.fq.gz␣\
␣␣Esc_col/input/illumina/test_100_400_2.fq.gz␣400␣0.01␣FR" \
  > reads.txt
perl GapFiller.pl -l reads.txt \
  -s Esc_col/output/dnaasm_without_gapfilling_from_unitigs.fa -T 16
cp standard_output/standard_output.gapfilled.final.fa \
  Esc_col/output/dnaasm_without_gapfilling_from_unitigs_gapfiller.fa

abyss-sealer -k55 -o test \
  -S Esc_col/output/dnaasm_without_gapfilling_from_unitigs.fa \
  Esc_col/input/illumina/test_100_400_1.fq.gz \
  Esc_col/input/illumina/test_100_400_2.fq.gz
cp test_scaffold.fa \
  Esc_col/output/dnaasm_without_gapfilling_from_unitigs_sealer.fa

cd /tmp
printf "max_rd_len=100\n[LIB]\navg_ins=400\nreverse_seq=0\nasm_flags=3\n␣\
␣␣rd_len_cutoff=100\nrank=1\npair_num_cutoff=3\nmap_len=32\n␣\
␣␣q1=Esc_col/input/illumina/test_100_400_1.fq.gz\n␣\
␣␣q2=Esc_col/input/illumina/test_100_400_2.fq.gz" > reads.txt
```

```
GapCloser -a Esc_col/output/dnaasm_without_gapfilling_from_unitigs.fa \
  -b reads.txt -o results.fa -t 16
cp results.fa \
  Esc_col/output/dnaasm_without_gapfilling_from_unitigs_gapcloser.fa
```

Finally, we evaluated resultant DNA sequences in terms of tandem repeats occurrence:

```
./trf409 Esc_col/ref.fa 1 100 100 80 10 250 2000 -l 6
./trf409 Esc_col/output/abyssUnitigsFromPET_1000.fa \
  1 100 100 80 10 250 2000 -l 6
./trf409 Esc_col/output/dnaasm_without_gapfilling_from_unitigs.fa \
  1 100 100 80 10 250 2000 -l 6
./trf409 Esc_col/output/dnaasm_with_gapfilling_from_unitigs.fa \
  1 100 100 80 10 250 2000 -l 6
./trf409 Esc_col/output/dnaasm_without_gapfilling_from_unitigs_gapcloser.fa\
  1 100 100 80 10 250 2000 -l 6
./trf409 Esc_col/output/dnaasm_without_gapfilling_from_unitigs_sealer.fa \
  1 100 100 80 10 250 2000 -l 6
./trf409 Esc_col/output/dnaasm_without_gapfilling_from_unitigs_gapfiller.fa\
  1 100 100 80 10 250 2000 -l 6
```

2.2.2 *Saccharomyces cerevisiae*

Firstly, we generated set of short reads by pIRS application:

```
cd pIRS_111
./pirs simulate -i Sac_cer/ref.fa -e 0.01 -a 1 -o test -x 50 -m 400 -v 40
mv test_100_400_* Sac_cer/input/illumina/
```

Then, we generated set of long reads by NanoSim application:

```
cd NanoSim/src
./simulator.py linear -r Sac_cer/ref.fa -c ecoli -o simulated -n 48500 \
  --min_len 1000 --max_len 10000 --perfect
mv simulated_reads.fasta Sac_cer/input/nanopore
```

After that, we assembled set of short reads by ABySS tool and removed unitigs shorter than 1 kbp:

```
cd abyss
abyss-pe name=org k=55 in='Sac_cer/input/illumina/test_100_400_1.fq.gz \
  Sac_cer/input/illumina/test_100_400_2.fq.gz'
mv org-3.fa Sac_cer/output/abyssUnitigsFromPET.fa
cd Sac_cer/output/
python fastaLengthFilter.py abyssUnitigsFromPET.fa 1000 100000000 \
  abyssUnitigsFromPET_1000.fa
```

Then, we linked contigs by dnaasm-link tool without gap filling:

```
./dnaasm -scaffold \
  -contigs_file_path Sac_cer/output/abyssUnitigsFromPET_1000.fa \
  -long_reads_file_path Sac_cer/input/nanopore/simulated_reads.fasta \
  -min_contig_length 0 -gapfilling 0 -min_reads 5 -min_lpr 5 -max_ratio 0.3\
```

```
  -kmer_size 15 -min_links 5 -distance 1000 -step 1
mv out out_1000
./dnaasm -scaffold -contigs_file_path out_1000 \
  -long_reads_file_path Sac_cer/input/nanopore/simulated_reads.fasta \
  -min_contig_length 0 -gapfilling 0 -min_reads 5 -min_lpr 5 -max_ratio 0.3\
  -kmer_size 15 -min_links 5 -distance 2000 -step 1
mv out out_2000
./dnaasm -scaffold -contigs_file_path out_2000 \
  -long_reads_file_path Sac_cer/input/nanopore/simulated_reads.fasta \
  -min_contig_length 0 -gapfilling 0 -min_reads 5 -min_lpr 5 -max_ratio 0.3\
  -kmer_size 15 -min_links 5 -distance 3000 -step 1
mv out out_3000
./dnaasm -scaffold -contigs_file_path out_3000 \
  -long_reads_file_path Sac_cer/input/nanopore/simulated_reads.fasta \
  -min_contig_length 0 -gapfilling 0 -min_reads 5 -min_lpr 5 -max_ratio 0.3\
  -kmer_size 15 -min_links 5 -distance 4000 -step 1
mv out out_4000
./dnaasm -scaffold -contigs_file_path out_4000 \
  -long_reads_file_path Sac_cer/input/nanopore/simulated_reads.fasta \
  -min_contig_length 0 -gapfilling 0 -min_reads 5 -min_lpr 5 -max_ratio 0.3\
  -kmer_size 15 -min_links 5 -distance 5000 -step 1
mv out out_5000
./dnaasm -scaffold -contigs_file_path out_5000 \
  -long_reads_file_path Sac_cer/input/nanopore/simulated_reads.fasta \
  -min_contig_length 0 -gapfilling 0 -min_reads 5 -min_lpr 5 -max_ratio 0.3\
  -kmer_size 15 -min_links 5 -distance 6000 -step 1
mv out out_6000
./dnaasm -scaffold -contigs_file_path out_6000 \
  -long_reads_file_path Sac_cer/input/nanopore/simulated_reads.fasta \
  -min_contig_length 0 -gapfilling 0 -min_reads 5 -min_lpr 5 -max_ratio 0.3\
  -kmer_size 15 -min_links 5 -distance 7000 -step 1
mv out out_7000
./dnaasm -scaffold -contigs_file_path out_7000 \
  -long_reads_file_path Sac_cer/input/nanopore/simulated_reads.fasta \
  -min_contig_length 0 -gapfilling 0 -min_reads 5 -min_lpr 5 -max_ratio 0.3\
  -kmer_size 15 -min_links 5 -distance 8000 -step 1
mv out out_8000
./dnaasm -scaffold -contigs_file_path out_8000 \
  -long_reads_file_path Sac_cer/input/nanopore/simulated_reads.fasta \
  -min_contig_length 0 -gapfilling 0 -min_reads 5 -min_lpr 5 -max_ratio 0.3\
  -kmer_size 15 -min_links 5 -distance 9000 -step 1
mv out out_9000
./dnaasm -scaffold -contigs_file_path out_9000 \
  -long_reads_file_path Sac_cer/input/nanopore/simulated_reads.fasta \
  -min_contig_length 0 -gapfilling 0 -min_reads 5 -min_lpr 5 -max_ratio 0.3\
  -kmer_size 15 -min_links 5 -distance 10000 -step 1
bbmap/rename.sh in=out out=out.fa
mv out.fa Sac_cer/output/dnaasm_without_gapfilling_from_unitigs.fa
```

Then, we linked contigs by dnaasm-link tool with gap filling:

```
./dnaasm -scaffold \
  -contigs_file_path Sac_cer/output/abyssUnitigsFromPET_1000.fa \
  -long_reads_file_path Sac_cer/input/nanopore/simulated_reads.fasta \
  -min_contig_length 0 -gapfilling 1 -min_reads 5 -min_lpr 5 -max_ratio 0.3\
  -kmer_size 15 -min_links 5 -distance 1000 -step 1
mv out out_1000
./dnaasm -scaffold -contigs_file_path out_1000 \
  -long_reads_file_path Sac_cer/input/nanopore/simulated_reads.fasta \
  -min_contig_length 0 -gapfilling 1 -min_reads 5 -min_lpr 5 -max_ratio 0.3\
  -kmer_size 15 -min_links 5 -distance 2000 -step 1
mv out out_2000
./dnaasm -scaffold -contigs_file_path out_2000 \
  -long_reads_file_path Sac_cer/input/nanopore/simulated_reads.fasta \
  -min_contig_length 0 -gapfilling 1 -min_reads 5 -min_lpr 5 -max_ratio 0.3\
  -kmer_size 15 -min_links 5 -distance 3000 -step 1
mv out out_3000
./dnaasm -scaffold -contigs_file_path out_3000 \
  -long_reads_file_path Sac_cer/input/nanopore/simulated_reads.fasta \
  -min_contig_length 0 -gapfilling 1 -min_reads 5 -min_lpr 5 -max_ratio 0.3\
  -kmer_size 15 -min_links 5 -distance 4000 -step 1
mv out out_4000
./dnaasm -scaffold -contigs_file_path out_4000 \
  -long_reads_file_path Sac_cer/input/nanopore/simulated_reads.fasta \
  -min_contig_length 0 -gapfilling 1 -min_reads 5 -min_lpr 5 -max_ratio 0.3\
  -kmer_size 15 -min_links 5 -distance 5000 -step 1
mv out out_5000
./dnaasm -scaffold -contigs_file_path out_5000 \
  -long_reads_file_path Sac_cer/input/nanopore/simulated_reads.fasta \
  -min_contig_length 0 -gapfilling 1 -min_reads 5 -min_lpr 5 -max_ratio 0.3\
  -kmer_size 15 -min_links 5 -distance 6000 -step 1
mv out out_6000
./dnaasm -scaffold -contigs_file_path out_6000 \
  -long_reads_file_path Sac_cer/input/nanopore/simulated_reads.fasta \
  -min_contig_length 0 -gapfilling 1 -min_reads 5 -min_lpr 5 -max_ratio 0.3\
  -kmer_size 15 -min_links 5 -distance 7000 -step 1
mv out out_7000
./dnaasm -scaffold -contigs_file_path out_7000 \
  -long_reads_file_path Sac_cer/input/nanopore/simulated_reads.fasta \
  -min_contig_length 0 -gapfilling 1 -min_reads 5 -min_lpr 5 -max_ratio 0.3\
  -kmer_size 15 -min_links 5 -distance 8000 -step 1
mv out out_8000
./dnaasm -scaffold -contigs_file_path out_8000 \
  -long_reads_file_path Sac_cer/input/nanopore/simulated_reads.fasta \
  -min_contig_length 0 -gapfilling 1 -min_reads 5 -min_lpr 5 -max_ratio 0.3\
  -kmer_size 15 -min_links 5 -distance 9000 -step 1
mv out out_9000
./dnaasm -scaffold -contigs_file_path out_9000 \
```

```
  -long_reads_file_path Sac_cer/input/nanopore/simulated_reads.fasta \
  -min_contig_length 0 -gapfilling 1 -min_reads 5 -min_lpr 5 -max_ratio 0.3\
  -kmer_size 15 -min_links 5 -distance 10000 -step 1
bbmap/rename.sh in=out out=out.fa
mv out.fa Sac_cer/output/dnaasm_with_gapfilling_from_unitigs.fa
```

Then, we filled gaps by GapFiller, Sealer and SOAPdenovo2 GapCloser tools:

```
cd GapFiller_v1-10_linux-x86_64
printf "lib_1 bwa Sac_cer/input/illumina/test_100_400_1.fq.gz \
  Sac_cer/input/illumina/test_100_400_2.fq.gz 400 0.01 FR" \
  > reads.txt
perl GapFiller.pl -l reads.txt \
  -s Sac_cer/output/dnaasm_without_gapfilling_from_unitigs.fa -T 16
cp standard_output/standard_output.gapfilled.final.fa \
  Sac_cer/output/dnaasm_without_gapfilling_from_unitigs_gapfiller.fa

abyss-sealer -k55 -o test \
  -S Sac_cer/output/dnaasm_without_gapfilling_from_unitigs.fa \
  Sac_cer/input/illumina/test_100_400_1.fq.gz \
  Sac_cer/input/illumina/test_100_400_2.fq.gz
cp test_scaffold.fa \
  Sac_cer/output/dnaasm_without_gapfilling_from_unitigs_sealer.fa

cd /tmp
printf "max_rd_len=100\n[LIB]\navg_ins=400\nreverse_seq=0\nasm_flags=3\n \
  rd_len_cutoff=100\nrank=1\npair_num_cutoff=3\nmap_len=32\n \
  q1=Sac_cer/input/illumina/test_100_400_1.fq.gz\n \
  q2=Sac_cer/input/illumina/test_100_400_2.fq.gz" > reads.txt
GapCloser -a Sac_cer/output/dnaasm_without_gapfilling_from_unitigs.fa \
  -b reads.txt -o results.fa -t 16
cp results.fa \
  Sac_cer/output/dnaasm_without_gapfilling_from_unitigs_gapcloser.fa
```

Finally, we evaluated resultant DNA sequences in terms of tandem repeats occurrence:

```
./trf409 Sac_cer/ref.fa 1 100 100 80 10 250 2000 -l 6
./trf409 Sac_cer/output/abyssUnitigsFromPET_1000.fa \
  1 100 100 80 10 250 2000 -l 6
./trf409 Sac_cer/output/dnaasm_without_gapfilling_from_unitigs.fa \
  1 100 100 80 10 250 2000 -l 6
./trf409 Sac_cer/output/dnaasm_with_gapfilling_from_unitigs.fa \
  1 100 100 80 10 250 2000 -l 6
./trf409 Sac_cer/output/dnaasm_without_gapfilling_from_unitigs_gapcloser.fa\
  1 100 100 80 10 250 2000 -l 6
./trf409 Sac_cer/output/dnaasm_without_gapfilling_from_unitigs_sealer.fa \
  1 100 100 80 10 250 2000 -l 6
./trf409 Sac_cer/output/dnaasm_without_gapfilling_from_unitigs_gapfiller.fa\
  1 100 100 80 10 250 2000 -l 6
```

3 Time and memory usage

Firstly, we trimmed *Caenorhabditis elegans* reference genome to first 1 Mbp:

```
cd time_eval/1M
head -c 1000000 ../ref.fa > ref.fa
```

Then, we generated set of short reads by pIRS application:

```
cd pIRS_111
./pirs simulate -i time_eval/1M/ref.fa -e 0.01 -a 1 -o test \
  -x 30 -m 400 -v 40
mv test_100_400_* time_eval/1M/input/illumina/
```

After that, we assembled set of short reads by ABySS tool and removed scaffolds shorter than 1 kbp:

```
cd abyss
abyss-pe name=org k=55 \
  in='time_eval/1M/input/illumina/test_100_400_1.fq.gz \
      time_eval/1M/input/illumina/test_100_400_2.fq.gz'
mv org-8.fa time_eval/1M/output/abyssScaffoldsFromPET.fa
cd time_eval/1M/output
python fastaLengthFilter.py abyssScaffoldsFromPET.fa 1000 100000000 \
  abyssScaffoldsFromPET_1000.fa
```

Then, we generated set of long reads by NanoSim application:

```
cd NanoSim/src
./simulator.py linear -r time_eval/1M/ref.fa -c ecoli -o simulated \
  -n 2000 --min_len 1000 --max_len 10000
mv sim_reads.fasta time_eval/1M/input/nanopore/sim_reads_10x.fasta
```

Lastly, we linked contigs by LINKS, SSPACE-LongRead and dnaasm-link tools:

```
cd links_v1.8.5
echo 'time_eval/1M/input/nanopore/sim_reads_10x.fasta' > nano_reads
perl LINKS.pl -f time_eval/1M/output/abyssScaffoldsFromPET_1000.fa
  -s nano_reads -z 1

cd SSPACE-LongRead_v1-1
perl SSPACE-LongRead.pl
  -c time_eval/1M/output/abyssScaffoldsFromPET_1000.fa
  -p time_eval/1M/input/nanopore/sim_reads_10x.fasta -b results -k 1

cd /home/wkusmir/dnaasm
export LD_LIBRARY_PATH=lib
./dnaasm -scaffold
  -contigs_file_path time_eval/1M/output/abyssScaffoldsFromPET_1000.fa
  -long_reads_file_path time_eval/1M/input/nanopore/sim_reads_10x.fasta
  -min_contig_length 0 -gapfilling 0
```
